# Total neoadjuvant therapy for locally advanced gastric cancer and esophagogastric junction adenocarcinoma: study protocol for a prospective, multicenter, single-arm, phase II clinical trial

**DOI:** 10.1186/s12876-022-02440-5

**Published:** 2022-07-28

**Authors:** Jinming Shi, Ning Li, Yuan Tang, Liming Jiang, Lin Yang, Shulian Wang, Yongwen Song, Yueping Liu, Hui Fang, Ningning Lu, Shunan Qi, Bo Chen, Ziyu Li, Shixin Liu, Jun Wang, Wenling Wang, Suyu Zhu, Jialin Yang, Yexiong Li, Dongbing Zhao, Jing Jin

**Affiliations:** 1 Department of Radiation Oncology, National Cancer Center/National Clinical Research Center for Cancer/ Cancer Hospital, Chinese Academy of Medical Sciences and Peking Union Medical College, Beijing, China; 2 State Key Laboratory of Molecular Oncology and Department of Radiology, National Cancer Center/National Clinical Research Center for Cancer/Cancer Hospital, Chinese Academy of Medical Sciences and Peking Union Medical College, Beijing, China; 3 State Key Laboratory of Molecular Oncology and Department of Medical Oncology, National Cancer Center/National Clinical Research Center for Cancer/Cancer Hospital, Chinese Academy of Medical Sciences and Peking Union Medical College, Beijing, China; 4 Key Laboratory of Carcinogenesis and Translational Research (Ministry of Education/Beijing), Department of Radiation Oncology, Peking University Cancer Hospital and Institute, Beijing, China; 5 Department of Radiation Oncology, Jilin Provincial Cancer Hospital, Changchun, China; 6 Department of Radiation Oncology, Hebei Provincial Cancer Hospital, Shijiazhuang, China; 7 Department of Radiation Oncology, Guizhou Provincial Cancer Hospital, Guiyang, China; 8 Department of Radiation Oncology, Hunan Cancer Hospital and Affiliated Cancer Hospital of Xiangya School of Medicine, Changsha, China; 9 Department of Radiation Oncology, Sichuan Provincial Cancer Hospital, Chengdu, China; 10 Department of Pancreatic and Gastric Surgery, National Cancer Center/National Clinical Research Center for Cancer/ Cancer Hospital, Chinese Academy of Medical Sciences and Peking Union Medical College, Beijing, China; 11 Department of Radiation Oncology, National Cancer Center/National Clinical Research Center for Cancer/Cancer Hospital and Shenzhen Hospital, Chinese Academy of Medical Sciences and Peking Union Medical College, Shenzhen, China

**Keywords:** Locally advanced, Gastric cancer, Esophagogastric junction adenocarcinoma, Neoadjuvant chemoradiotherapy, Consolidated chemotherapy

## Abstract

**Background:**

Gastric cancer ranks high in terms of morbidity and mortality worldwide. Multimodal therapy is therefore essential for locally advanced gastric cancer. Recent studies have demonstrated that both perioperative chemotherapy and neoadjuvant chemoradiotherapy can improve the prognosis of patients. However, the completion rate of chemotherapy after surgery remains low, which may affect survival. Thus, identifying the best way to combine radiotherapy, chemotherapy and surgery is important. The aim of this study was to explore the toxicity and efficacy of the total neoadjuvant therapy modality for locally advanced gastric cancer.

**Methods:**

This study will be a prospective, multicenter, single-arm, phase II clinical trial. Patients diagnosed with locally advanced (stage cT3-4 and cN positive, AJCC 8th) gastric cancer and gastroesophageal junction adenocarcinoma will be enrolled. Patients will initially receive radiotherapy (95% planned target volume: 45 Gy/25 f) and concurrent chemotherapy (S-1: 40–60 mg twice a day) followed by six cycles of consolidated chemotherapy (SOX, consisting of S-1 and oxaliplatin) and surgery. The primary objective will assess pathological complete response; the secondary objectives will include toxicities assessing surgical complications, the tumor downstaging rate and the R0 resection rate.

**Discussion:**

Investigation of total neoadjuvant therapy in gastric cancer is limited. The goal of this trial is to explore the efficacy and toxicity of total neoadjuvant therapy for locally advanced gastric cancer and gastroesophageal junction adenocarcinoma.

*Trial registration*: Clinicaltrials.gov (NCT04062058, August 20, 2019).

## Background

In recent years, the morbidity of gastric cancer (GC) has declined worldwide, while the percentage of esophagogastric junction (EGJ) adenocarcinoma has increased rapidly [1]. In China, GC still ranks second in terms of morbidity and mortality [2], with most patients diagnosed at a locally advanced stage. Although radical surgery is the main treatment approach for these patients, the risk of recurrence remains high after surgery [3], and the prognosis is extremely poor. Hence, the combination of radiotherapy, chemotherapy and surgery is essential. Attempts to combine these three important treatment methods remain a research hotspot; in the early years, radiotherapy and chemotherapy were mostly administered after surgery. The INT0116 study enrolled 556 patients diagnosed with GC, all of whom underwent radical surgery. The results showed that compared with surgery alone, adjuvant chemoradiotherapy significantly improved overall survival [4]. Gradually, as D2 resection has become more widespread, the value of adjuvant chemoradiotherapy has been challenged, especially after the results of the ARTIST 2 trial were published [5]. Recently, some phase II or III studies have verified the value of neoadjuvant radiotherapy for locally advanced GC and EGJ cancer in terms of local control and the R0 reaction rate [6, 7]. With advancements in neoadjuvant therapy, the MAGIC, FFCD and FLOT4 trials have confirmed that perioperative chemotherapy will improve patient prognosis [8–10]. Currently, both perioperative chemotherapy and preoperative concurrent chemoradiotherapy are recommended for patients with locally advanced GC according to the National Comprehensive Cancer Network (NCCN) guidelines [11]. However, the completion rate of therapy after surgery is lower than that before surgery, which may influence survival outcomes [12]. Therefore, total neoadjuvant therapy is a promising treatment modality. The POET study is the first phase III clinical trial comparing induced chemotherapy followed by neoadjuvant chemoradiotherapy (NCRT) and surgery with neoadjuvant chemotherapy (NCT). The study ultimately enrolled 119 patients due to slow enrollment. The pathological complete response (pCR) rates for NCRT and NCT were 14.3% and 1.9%, respectively (*P* = 0.03), and the 5-year overall survival rates were 39.5% and 24.4%, respectively (*P* = 0.055) [13]. NCRT showed a tendency to improve survival; however, the dose of radiotherapy was 30 Gy, and the radiotherapy machine and chemotherapy regimen were outdated. Based on the current advancements in intensity-modulated radiotherapy equipment and chemotherapy regimens, our study will assess a newly designed total neoadjuvant therapy comprising NCRT followed by consolidated chemotherapy and surgery for locally advanced GC and EGJ adenocarcinoma. The objective of this trial is to confirm the feasibility and safety of this treatment mode. We aim to provide additional clinical evidence to support the administration of new neoadjuvant treatments.

## Methods

### Study design and objectives

**Fig. 1:**
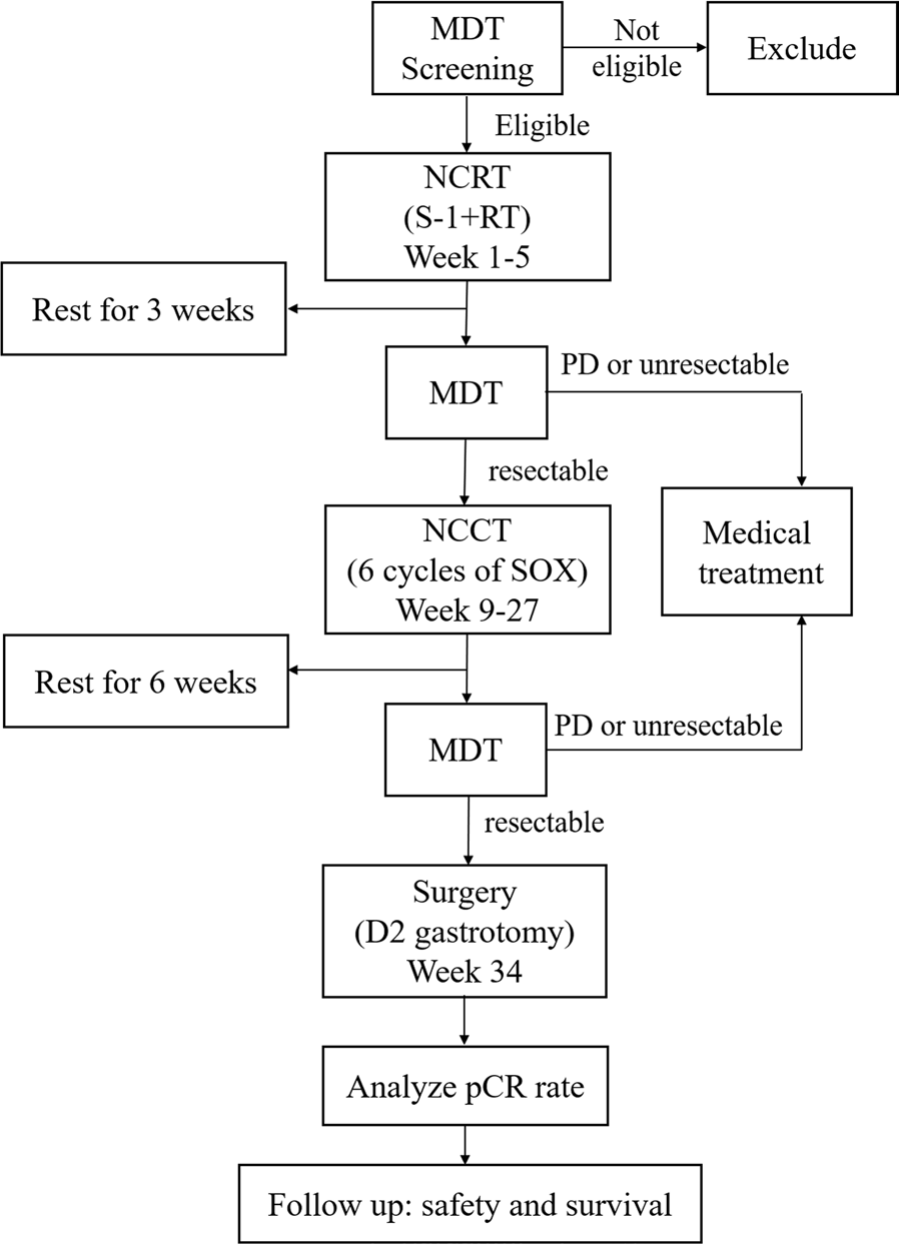
Flow chart. MDT: Multiple Disciplinary Team; NCRT (neoadjuvant chemoradiotherapy): 45 Gy in 25 fractions over 5 weeks concurrently with tegafur; NCCT (neoadjuvant consolidated chemotherapy): is consisted of 6 cycles of SOX regimen. pCR: pathological Complete Response

This study will be a single-arm, open-label and multicenter phase II trial. Patients diagnosed with locally advanced (stage cT3-4 and cN positive, AJCC 8th) GC or EGJ adenocarcinoma will be recruited. Before recruitment, each patient will receive gastroscopy, endoscopic ultrasound, and chest-abdomen-pelvis enhanced CT for tumor detection and stages. A multidisciplinary team will screen patients meeting the enrollment conditions. The clinical T stage will be identified by imaging features and laparoscopy information. Metastatic regional nodes will be considered based on their size and biological characteristics. After careful screening, patients will be initially treated with NCRT. The technique of Intensity-Modulated Radiation Therapy (IMRT) or Volumetric Modulated Arc Therapy (VMAT) will be adopted in this trial, with a dose of 45 Gy/25 f to the planned target volume (PTV), and the concurrent chemotherapy will be S-1. Then, the patients will begin six courses of neoadjuvant consolidated chemotherapy (NCCT) using the S-1 and oxaliplatin (SOX) regimen. Finally, patients will undergo surgery after total neoadjuvant therapy. An overview of the study design is shown in Fig. 1.

The primary objective is the assessment of a pathological complete response (pCR), which is defined as no tumor cells in the primary tumor and metastatic regional lymph node. Secondary objectives include assessing toxicities during NCRT and NCCT, surgical complications, the tumor downstaging rate and the R0 resection rate. Recruitment began in November 2019 and is expected to be finished in September 2023. Seven centers in China are participating in this study. The protocol was approved by the institutional ethics committees of these centers and was registered at ClinicalTrials.gov with registration number NCT04062058.

### Eligibility criteria

In this multicenter phase II clinical trial, patients aged 18–70 years with Karnofsky performance status (KPS) ≥ 70; histologically proven gastric cancer or Siewert II/III EGJ adenocarcinoma [14]; and clinical stage T3–4 and N positive without distant metastases were enrolled. No other anticancer treatments were allowed before enrollment. Laboratory investigation requirements were as follows: white blood cell (WBC) count ≥ 3.0 × 10^9^/L, platelet count (PLT) ≥ 100 × 10^9^/L, hemoglobin count (Hb) ≥ 10 g/dl, neutrophil count ≥ 1.5 × 10^9^/L; total bilirubin ≤ 1.5 times the upper limit of normal (ULN); and aspartate aminotransferase (AST) and alanine aminotransferase (ALT) ≤ 1.5 × ULN; serum creatinine (SCr) ≤ 1.0 × ULN; alkaline phosphatase (AKP) ≤ 2.5 × ULN. Patients agreed to sign an informed consent form before enrollment.

The exclusion criteria included patients aged more than 70 years or less than 18 years, prior anticancer treatment, pregnant or lactating females, metastatic disease, history of myocardial infarction or cerebral infarction within the past 6 months, active infections of tuberculosis or hepatitis, and refusal to provide informed consent.

### Treatment

Total NCRT will involve NCRT followed by six cycles of consolidated chemotherapy and surgery.

#### Neoadjuvant chemoradiotherapy

Before the simulation, patients will be instructed to fast for at least 4 h and drink 300 ml semifluid diet 10 min before CT (4DCT) simulation and daily radiotherapy to maintain a consistent stomach volume. The patients will lie on the table in the supine position with their arms crossed above their heads and will be stabilized by a thermoplastic mask under free-breathing conditions. CT scanning will be performed from the clavicle to the fifth lumbar spine, with a scanning thickness of 0.5 cm. IMRT or VMAT will be recommended during radiotherapy. The 45 Gy dose of radiotherapy will be delivered in 25 fractions. Concurrent chemotherapy is S-1 administered at a dose of 40 to 60 mg twice daily according to body surface area (BSA) (Table 1).

**Table 1:** Dose of S-1

BSA (m^2^)	Dose
< 1.25 m^2^	40 mg bid
1.25—1.5 m^2^	50 mg bid
≥ 1.5 m^2^	60 mg bid

Based on the EORTC-ROG expert opinion and third edition of the Japan Gastric Cancer Association guideline [15, 16], the radiation field will include the gross tumor, metastatic regional nodes and elective regional lymph node stations. The gross tumor volume (GTV) will be defined as a primary tumor determined by available technology. The diagnosis of metastatic regional lymph nodes will require comprehensive judgment based on their size and morphology. For example, for metastatic regional lymph nodes, the short axis diameter of the lymph nodes should be larger than 8 mm; a rounded shape, necrosis in the center of the lymph node, and clustered lymph nodes should be observed; marked enhancement should be identified during enhanced CT; and lymph nodes should be enlarged significantly during the examination. A metastatic regional lymph node will be defined as a metastatic regional node (GTVnd). The clinical target volume (CTV) contains GTV, GTVnd and elective regional lymph node stations according to the location of the tumor (Table 2).

**Table 2:** Elective lymph node station according to tumor location

Tumor location	Elective lymph node stations
EGJ (Siewert II)	1, 2, 3, 4sa, 7, 9, 11p, 19, 20, 110, 111
EGJ (Siewert III)	1, 2, 3, 4sa, 7, 9, 10, 11p, 11d, 19, 20, 110, 111
GC (proximal third)	1, 2, 3, 4sa, 4sb, 7, 9, 10, 11p, 11d, 19
GC (middle third)	1, 2, 3, 4sa, 4sb, 4d, 7, 8, 9, 10, 11p, 11d, 18, 19
GC (distal third)	3, 4d, 5, 6, 7, 8, 9, 11p, 11d, 12, 13, 17, 18

Referring to the ICRU-62 report [17], the planned target volume will be derived from CTV plus 1 cm in the cranio-caudal direction and 0.5 to 0.7 cm in the anterior–posterior and left–right directions. The dose limitations of the organs at risk (OARs) will be as follows: Dmax stomach ≤ 50 Gy, V30 liver < 26%, V20 kidney < 26%, Dmean kidney < 18 Gy, Dmax spinal cord PRV ≤ 40 Gy, V10 bone marrow < 26%, Dmax intestine ≤ 42 Gy, and V40 intestine < 10% (for EGJ cancer, heart and lung should be included, V30 heart < 26%, and V20 lung < 20%).

Cone beam computed tomography (CBCT) scans will be performed daily during the first week and then once a week at least based on the clinical situation. Routine blood tests will be monitored weekly, and biochemical tests will be performed every two weeks during NCRT. Patients will take a 3-week break, and then a physical examination will be conducted before NCCT. The contents of the examination are shown in Table 3.

**Table 3:** Assessment and intervention schedule for the trial

	Screening	During NCRT	Before NCT	During NCT	Before surgery	Follow-up		
						Year 1-2: every 3 months	Year 3-5: every 6 months	> 5 year: every 1 year
Physical examination	√	X	√	X	√	√	√	√
Blood routine	√	X	√	X	√	√	√	√
Urine routine	√				√			
Stool routine	√				√			
Biochemical test	√	△	√	△	√	√	√	√
CEA	√		√	○	√	√	√	√
CA199	√		√	○	√	√	√	√
CA724	√		√	○	√	√	√	√
Electrocardiogram	√				√			
Enhanced CT*	√		√	○	√	√	√	√
Ultrasound**	√		√	○	√	√	√	√
Gastroscopy	√				√	√	√	√
Endoscopic ultrasound	√				√			
Pathology	√							

√: once, X: weekly, △: biweekly, ○: every 3 cycles

*scanning range: chest, abdomen and pelvis

**scanning range: neck and supraclavicular

#### Neoadjuvant consolidated chemotherapy

The NCCT regimen will involve six cycles of SOX, with each cycle lasting for 21 days. Oxaliplatin will be delivered intravenously at a dose of 130 mg/m2 on day 1, and S-1 will be given orally at a dosage of 40 to 60 mg twice daily according to BSA (Table 1) on days 1 to 14. Patients will take a seven-day break after 14 days of chemotherapy, and complete physical examination will be performed every 3 cycles.

#### Surgery

Standard radical surgery will be performed 6 weeks after the last cycle of chemotherapy. For the type of resection, laparoscopic will be recommended. For the Siewert II EGJ, laparoscopy can also be combined with thoracoscopy. For the proximal GC and EGJ, esophagectomy and total or subtotal gastrectomy will be recommended. For the middle or distal GC, subtotal gastrectomy will be recommended. For the gastrointestinal tract reconstruction, Billroth I or Billroth II reconstruction will be used for subtotal gastrectomy and Roux-en-Y reconstruction will be used for total gastrectomy. D2 resection will be recommended. The definition of D2 lymph node dissection can be referred to NCCN guidelines [11]. At least 16 or greater lymph nodes are required to examine during the surgery. If the disease progresses during neoadjuvant therapy, a multiple disciplinary team (MDT) will be required to discuss the subsequent treatment.

### Tumor response and toxicity criteria

The contents of the tumor efficacy assessments during therapy and follow-up are listed in Table 3. Response Evaluation Criteria in Solid Tumors (RECIST) version 1.1 guidelines will be adopted as the tumor response evaluation criteria [18]. Toxicities occurring during therapy will be assessed based on Common Terminology Criteria of Adverse Events (CTCAE) version 3.0. It will be necessary to reduce the dose or to stop treatment when serious adverse effects occur. During neoadjuvant or concurrent chemotherapy, when grade III gastrointestinal, neurotoxic or grade IV leukopenia occurs without recovery within 5 days after treatment, the dose will be reduced to 80% of the original dose. Chemotherapy will be stopped if any grade IV adverse effects occur except leukopenia. Radiotherapy will be stopped if a patient does not recover in 7 days when grade III gastrointestinal occurs after treatment. Any grade IV adverse effect except leukopenia will result in termination of radiotherapy. When a patient recovers to grade 0-I, radiotherapy may continue without receiving concurrent chemotherapy. It will be unnecessary to adjust the radiation dose. Surgical complications will be defined as anastomotic leakage, anastomotic bleeding or abdominal infection and others within 30 days after surgery. Tumor pathological response will be assessed by using the tumor response grading (TRG) system proposed by Mandard et al. [19]. All adverse events will be recorded in a case report form (CRF). Adverse events will be reported to the ethical institution committee within 24 h and dealt with immediately.

### Sample size calculation

The primary objective is to determine the pCR rate in this trial. According to the results of the prospective phase II trial previously conducted at our center [20], the pCR rate of patients who received NCT was 14% (P0). We assume that after NCRT and six cycles of NCCT, the pCR rate will reach 28% in this study. Taking into consideration the loss of patients and the optimal two-stage design of phase II clinical trials, the test level α is 0.05 with a power of 80%. The required sample size of the study will be 82 patients. After 33 patients are enrolled, a planning interim analysis will be conducted. If the number of patients who received pCR is less than five, the study will be closed.

### Statistical analysis

For descriptive statistics, categorical variables are described by frequency, rate, or 95% confidence interval. Continuous variables are described by the mean, median, quartile or standard deviation. For comparisons between groups, categorical variables will be compared by using the chi-square test or Fisher’s exact test. For continuous variables, the paired *t* test or Wilcoxon test will be used to compare changes between baseline and endpoint. The Kaplan–Meier method will be used to predict overall survival probabilities at specific time points. All statistical tests will be two-sided. The level of significance will be set to *P* < 0.05.

### Follow-up

After completing the treatment according to the protocol, patients will receive the examinations listed in Table 3. Patients will be examined every 3 months in the first 2 years, every 6 months during years 3 to 5, and then once a year after 5 years. During follow-up, investigators will collect information on surgical complications, late toxicity, other treatments, and recurrence and survival.

### Quality assurance

The quality assurance (QA) team associated with this study will include experienced experts in the field of radiotherapy, dosimetry, medical physics, medicine, surgery, imaging, pathology, and data censors. Diagnostic images before and after neoadjuvant treatment will be reviewed centrally and independently by two radiologists. The first three cases of target volume delineation from each center will be reviewed centrally and then checked randomly. Data censors in this study will stay in communication with branch-centers and check the quality of the data collection randomly.

### Data collection and management

Every center needs at least one physician to be responsible for enrolling patients in this trial and arranging patient therapy. Two physicians will collect data and complete the CRF at their center. When modifying data, the researcher will need to sign their name and record the date of modification on the CRF. The CRF will be regarded as raw material, and all data in the CRF will be archived electronically on a specific computer. All electronic documents will be confidential, and only the data manager will have the password. Only the project leader will have the right to use the database, and other researchers will not be allowed to use the database unless permitted.

## Discussion

GC ranks high in terms of morbidity worldwide, and multidisciplinary treatments are urgently needed, particularly for locally advanced GC. Recent published or ongoing clinical trials associated with NCRT or NCT are listed in Table 4. In our study, the objective will be to explore the short-term efficacy and safety of total neoadjuvant therapy for locally advanced GC and EGJ adenocarcinoma. The results may offer more therapeutic options for physicians to offer these patients.

**Table 4:** Summary of published or ongoing prospective clinical trials associated with NCRT or NCT in EGJ/GC adenocarcinoma

Category	Author/year	No. of pts	Tumor stage	Study design	RT dose	Concurrent CT regime	CT regimes	Finding/ Clinical trial registration number
Neoadjuvant radiotherapy	Shapiro [7]	366	T2–3N0/N +	NCRT + S vs S	41.4 Gy	TC	/	7y OS: 41%vs28% (*P* < 0.05)
	Tian [21]	150	T2-4N0/N +	NCRT + S vs S	45 Gy	XELOX	/	3yOS:63.4% vs 52.2%(*P* = 0.019)
	Stahl [13]	126	cT3-4NxM0	NCT + NCRT + S vs NCT + S	30 Gy	EP	PF	5yOS:39.5% vs 24.4%(*P* = 0.055)
	Liu [22]	36	T4aN + /T4b	NCT + NCRT + S + CT	45 Gy	S-1	SOX	2y:56%
	Kim [23]	42	T3-4N0/N +	NCT + NCRT + S + CT	45 Gy	S-1	SOX	3y:75.5%
Perioperative chemotherapy	Cunningham [9]	503	T1-4N0/N +	NCT + S + CT vs S	/	/	ECF	5yOS:36.3%vs23% (*P* = 0.009)
	Ychou [10]	224	T1-4N0/N +	NCT + S + CT vs S	/	/	CF	5yOS: 38%vs24%(*P* = 0.02)
	Al-Batran [8]	716	T2-4N0/N +	NCT + S + CT vs NCT + S + CT	/	/	FLOT vs ECF/ECX	5yOS:45%vs36% (*P* = 0.012)
	Zhang [24]	1094	cT4aN + /cT4b	NCT + S + CT vs S + CT vs S + CT	/	/	SOX(Peri) vs SOX(post) vs XELOX(post)	3yDFS:59.4% vs 56.5% vs 51.1%(arm1 vs arm3:*P* = 0·028)
Ongoing	TOPGEAR [25]	752	T3-4N0/N +	NCT + NCRT + S + CT vs NCT + S + CT	45 Gy	5-Fu/Cap	ECF	NCT01924819
	PREACT [26]	682	T3-4aN + /T4b	NCT + NCRT + CT vs NCT + S + CT	45 Gy	S-1	SOX	NCT03013010
	NEO-CRAG	620	T3N2-N3/T4aN + /T4b	NCT + NCRT + S + CT vs NCT + S + CT	45 Gy	XELOX	XELOX	NCT01815853
	CRITICS II [27]	207	T2-4N0/N +	NCT + NCRT + S vs NCRT + S vs NCT + S	45 Gy	TC	DOC	NCT02931890
	CRADLE	214	T3-4N0/N +	NCRT + S + CT vs S + CT	50 Gy	SOX	SOX	NCT02193594

The CROSS study was the first to report a significant survival benefit for the NCRT group compared with the surgery alone group among patients with EGJ adenocarcinoma [7]. With the development of NCT, the choice between NCRT and NCT has become controversial. The POET study was the first to compare NCRT with NCT and found that NCRT significantly downstaged tumor stage and improved the pCR rate [13]. One meta-analysis confirmed that NCRT can improve local control compared with NCT, but improvements in overall survival were not observed [28].

Currently, the value of NCRT for EGJ is clearly verified and recommended by the NCCN guidelines [11]. However, the value of NCRT for middle and distal GC is still lack of reliable evidence. The RTOG9904 study included 42 patients with GC and firstly found the value of NCRT in GC. The pCR rate was 26%, and the 1-year OS was 72% in this study [29]. Recently, Liu et al. included 40 patients with GC who received NCRT. The pCR rate was 13.9% and the medial survival time was 30.3 months [22]. Ahmed et al. included 32 patients with GC who received NCRT. The pCR rate was 18.8% and the 2-year OS was 51.3% [30]. Although, some phase II clinical trial has found the value of NCRT in middle and distal GC, it still needs evidence from larger sample size prospective clinical trials.

Perioperative therapy has advanced rapidly, and 6–8 cycles of perioperative chemotherapy are currently recommended according to the NCCN guidelines [11]. In the MAGIC study, six cycles of an ECF/ECX (stepirubicin, cisplatin and fluorouracil or capecitabine) regimen were used before and after surgery; although overall survival improved, only 41.6% of the patients completed the planned six cycles [9]. In the FLOT4 study, only 46% of patients completed all perioperative treatments [8]. In the CRITICS trial, the completion rate of adjuvant chemotherapy and chemoradiotherapy was 60% [12]. As mentioned above, the complete percentage of adjuvant therapy was quite low. Hence, in this trial, all perioperative treatment was administered before surgery. Moreover, preoperative chemotherapy may reduce the surgery rate for potential distant metastasis patients. In recent years, an increasing number of investigators have focused on the use of a total neoadjuvant therapy modality in patients with gastrointestinal tumors, especially those with rectal cancer [31], yet similar studies on GC are quite limited. Kim et al. explored the induction of NCT followed by NCRT for GC patients; adjuvant chemotherapy was needed for patients whose tumors were not downstaged to stage 0 to I after surgery. A total of 42 patients were enrolled in their study; 33.3% were pathologically downstaged to stage 0-I after preoperative treatment, and the 3-year overall survival rate was 75.5% [23]. The results of the use of total neoadjuvant therapy approaches for GC and EGJ adenocarcinoma phase II clinical trials were published at the 2019 ASTRO (American Society for Therapeutic Radiology and Oncology) meeting. Patients received 8 cycles of induction chemotherapy (FOLFIRINOX: Oxaliplatin, Irinotecan, Fluorouracil and leucovorin) followed by NCRT and surgery. Of the 25 patients enrolled in this trial, 20 underwent surgery, and the pCR rate was 35%. The results of this trial demonstrated the promising effects of total neoadjuvant therapy [32]. To further improve the downstaging rate, we prolonged the interval time between radiotherapy and surgery. The strategy of receiving NCRT followed by NCCT and surgery is rarely reported for GC.

We previously conducted a preliminary study to evaluate the efficacy of NCRT for locally advanced GC in Chinese patients. Among the 11 patients enrolled, two received the paclitaxel and carboplatin combined with radiotherapy. One patient experienced a serious adverse effect and concurrent chemotherapy interruption in the early stage. The other choked while eating half a month after chemoradiotherapy. The other nine patients received one drug with no serious reactions [33]. We moved all postoperative chemotherapy to before surgery considering toxicities, and we consider S-1 to be an appropriate choice when combined with radiotherapy.

Regarding the choice of NCT regimen, Sah et al. [34] compared FLOT (Oxaliplatin, Leucovorin, Fluorouracil and Docetaxel) and SOX regimens for locally advanced GC, with little difference in toxicity and prognosis. The SOX regimen has been widely used in Eastern clinical trials [24, 35, 36] and has been recommended in guidelines [11, 37]. Because our study is an exploratory study, six cycles of NCT are recommended according to the patients’ degree of tolerance. We previously designed a prospective study for GC and EGJ cancer. Patients who were diagnosed at clinical stage T3-4 or N positive were randomly assigned to the perioperative chemotherapy group or NCRT combined with surgery and adjuvant chemotherapy group. Surgery was performed 4–10 weeks after the completion of radiotherapy and chemotherapy. During the 2018 ASTRO meeting, we reported that the primary pCR rates in the NCRT and NCT groups were 14.3% and 11.1%, respectively (*P* = 0.724). The disease-free survival rate (87.1% vs. 63.9%, *P* = 0.05) and local tumor-free survival rate (100% vs. 73.3%, *P* = 0.014) in the NCRT group were better than those in the NCT group. No grade V reaction or perioperative death occurred in either group [20]. This research laid a methodological foundation and provided data to calculate the sample size in this study.

These study results are promising, but one limitation in our study is that we did not employ diagnostic laparoscopy for staging. To solve this problem, two imaging physicians with more than 10 years of working experience in gastrointestinal tumor diagnosis conducted tumor staging before enrollment.

To the best of our knowledge, this is the first attempt to treat GC or EGJ adenocarcinoma patients with NCRT followed by NCCT and surgery. We hope that the results of this promising total neoadjuvant therapy modality may provide a new strategy to further improve patient prognosis.

## Trial status

This study was registered online in August 2019. The recruitment started in November 2019 and is still ongoing.

## Data Availability

The datasets used or analyzed during the current study are available from the corresponding author on reasonable request.

## Abbreviations

ASTRO: American Society for Therapeutic Radiology and Oncology
BSA: Body surface area
4DCT: Four-dimensional computed tomography
CRF: Case report form
CT: Computed tomography
CTV: Clinical target volume
CTCAE: Common terminology criteria for adverse events
CBCT: Cone beam computed tomography
EGJ: Esophagogastric junction
ECF/ECX: Stepirubicin, cisplatin and fluorouracil or capecitabine
FOLFIRINOX: Oxaliplatin, irinotecan, fluorouracil and leucovorin
FLOT: Oxaliplatin, leucovorin, fluorouracil and docetaxel
GC: Gastric cancer
IMRT: Intensity-modulated radiation therapy
KPS: Karnofsky performance status
MDT: Multiple disciplinary team
NCRT: Neoadjuvant chemoradiotherapy
NCT: Neoadjuvant chemotherapy
NCCT: Neoadjuvant consolidated chemotherapy
NCCN: National comprehensive cancer network
OAR: Organs at risk
pCR: Pathological complete response
QA: Quality assurance
RECIST: Response evaluation criteria in solid tumors
SOX: Tegafur and oxaliplatin
TRG: Tumor response grading
VMAT: Volumetric modulated arc therapy
